# Genetic Ablation of the Inducible Form of Nitric Oxide in Male Mice Disrupts Immature Neuron Survival in the Adult Dentate Gyrus

**DOI:** 10.3389/fimmu.2021.782831

**Published:** 2021-12-01

**Authors:** Gabriel G. Fernandes, Karla C. M. Costa, Davi S. Scomparin, Juliana B. Freire, Francisco S. Guimarães, Alline C. Campos

**Affiliations:** Department of Pharmacology, Ribeirão Preto Medical School, University of São Paulo, Ribeirão Preto, Brazil

**Keywords:** iNOS, adult hippocampal neurogenesis, chronic stress, microglia, behavior

## Abstract

Inducible nitric oxide synthase (iNOS) is an enzyme upregulated in the brain during neuroimmune stimuli which is associated with an oxidative and pro-inflammatory environment in several brain regions, including the hippocampal formation and the prefrontal cortex. The dentate gyrus of the hippocampal formation is the site of a process known as adult hippocampal neurogenesis (AHN). Although many endogenous and extrinsic factors can modulate AHN, the exact participation of specific proinflammatory mediators such as iNOS in these processes remains to be fully elucidated. Here, we investigated how the total genetic ablation of iNOS impacts the hippocampal neurogenic niche and microglial phenotype and if these changes are correlated to the behavioral alterations observed in iNOS knockout (K.O.) mice submitted or not to the chronic unpredictable stress model (CUS - 21 days protocol). Contrary to our initial hypothesis, at control conditions, iNOS K.O. mice displayed no abnormalities on microglial activation in the dentate gyrus. However, they did exhibit impaired newborn cells and immature neuron survival, which was not affected by CUS. The reduction of AHN in iNOS K.O. mice was accompanied by an increased positive coping response in the tail suspension test and facilitation of anxiety-like behaviors in the novelty suppressed feeding. Next, we investigated whether a pro-neurogenic stimulus would rescue the neurogenic capacity of iNOS K.O. mice by administering in control and CUS groups the antidepressant escitalopram (ESC). The chronic treatment with ESC could not rescue the neurogenic capacity or the behavioral changes observed in iNOS K.O. mice. Besides, in the ventromedial prefrontal (vmPFC) cortex there was no change in the expression or the chronic activation of PV neurons (evaluated by double labeling PV with FOSB) in the prelimbic (PrL) or infralimbic subregions. FOSB expression, however, increased in the PrL of iNOS K.O. mice. Our results suggest that iNOS seems essential for the survival of newborn cells and immature neurons in the hippocampus and seem to partially explain the anxiogenic-like behavior observed in iNOS K.O. mice. On the other hand, the iNOS ablation appears to result in increased activity of the PrL which could explain the antidepressant-like behaviors of iNOS K.O mice.

## 1 Introduction

Adult hippocampal neurogenesis (AHN) consists of a well-orchestrated form of neuroplasticity present amongst several mammals, including humans (still on dabate), that involves symmetrical and asymmetrical division of neural stem cells (NSCs) present in the subgranular zone of the dentate gyrus, their migration towards the granular layer and their maturation and integration onto the local neurocircuitry (1–3).

Although their exact function remains obscure, several lines of evidence suggest that AHN is relevant for proper behavioral adaptations evoked by acute and chronic stressful experiences (4–6). Reciprocally, stress hormones and stress exposure are important negative modulators of AHN (7, 8). Besides stress hormones, preclinical studies suggest that inflammatory mediators may underlie the anti-neurogenic effects of stress (9, 10). Corroborating these premises, impaired AHN has been associated with the development of depressive and anxiety-like behaviors in rodents (11), and patients suffering from major depressive or anxiety disorders have altered levels of circulating cytokines (12–16).

During inflammatory states, some classes of cytokines upregulate the expression of the inducible form of nitric oxide synthase (iNOS). Furthermore, in contrast to the other isoforms of NOS, upon activation, this enzyme produces high NO levels from L-arginine, which creates a toxic environment due to its oxidative properties (17).

In preclinical studies, pharmacological or genetic manipulation of iNOS in rodents produce antidepressant-like behaviors in the forced swim test (FST) (18) anti-anhedonic-like behavior in chronic stress models (19). Noteworthy, iNOS is elevated in the peripheral blood of depressed patients (20) and polymorphisms linked to iNOS have been associated with an increased risk -factor for recurrent depressive disorder (21).

In the literature, several studies have linked intact AHN with better behavioral performances in models involving chronic stress. However, only a few studies have investigated the role of iNOS in adult neurogenesis. In this direction, Keilhoff (22) showed that iNOS knockout (K.O.) mice had an impaired proliferation rate of adult newborn neurons on the dentate gyrus, and this effect was not accompanied with a disrupted adult new-born neurons survival rate (22). Moreover, Carreira and colleagues (2014) showed that, after a proinflammatory stimulus, the proliferation of neural precursor cells decreased in another neurogenic niche, the subventricular zone (SVZ). In addition, the antiproliferative effects observed were mediated by iNOS present in microglia cells (23).

In the hippocampal neurogenic niche, microglia cells, the immunocompetent glia cell present throughout the central nervous system (24), have been identified as important players in the process of AHN by eliminating apoptotic new-born cells (25), releasing factors that may enhance (26) or diminish (27) rates of new-born cells proliferation and/or survival (28, 29). Of note, chronic stress exposure decreases AHN and recruits microglia cells (30–32) inducing phenotypic changes and the local production of inflammatory mediators and iNOS (26, 33, 34).

However, to the best of our knowledge, the link between behavioral changes induced by chronic stress, AHN and the participation of microglial cells in the hippocampal neurogenic niche remains to be elucidated. Therefore, in the present study, we test the hypothesis that genetic ablation of iNOS would prevent the neurogenic and behavioral effects of chronic stress by precluding microglia from assuming a proinflammatory phenotype. Surprisingly, our results refuted this initial hypothesis suggesting that iNOS K.O. mice had basal impaired AHN which was not negatively affected by CUS exposure, rescued by the treatment with antidepressant nor accompanied by microglia morphological changes. Also, iNOS K.O. mice displayed an active coping response and an anxiogenic-like phenotype prior CUS exposure. In addition to AHN, we also evaluate the pattern of activation of prelimbic and infralimbic portions of the ventromedial prefrontal cortex as well as parvalbumin (PV) interneurons in iNOS K.O mice (35–37). iNOS K.O. showed increased levels of FOSB, a transcription factor associated with neuronal recruitment and activation, in the prelimbic cortex (38), but no changes in the profile of activation of infralimbic or PV neurons. Therefore, the anxiogenic profile related to the facilitation of acute stress coping strategies observed in iNOS K.O. mice may reflect distinct cellular modifications (impaired AHN coupled with increased prelimbic activity).

## 2 Material and Methods

### 2.1 Animals

All experiments involving animals were conducted following the ARRIVE guidelines (39). Animal procedures were previously approved by the Experimental Committee of the University of São Paulo (protocol number 182/2017), which conforms to the Brazilian College of Animal Experimentation (COBEA). Male iNOS knockout (iNOS K.O.) and W.T. C57BL6 mice were acquired from the Special Mice Breeding Center and Central Animal House Facility of the University of São Paulo, Ribeirão Preto, respectively. For outbred experiments, 28 iNOS K.O. and 26 W.T.mice were obtained with matching ages and left undisturbed until 8-10 weeks old when they were used in experimental protocols. For the inbred experiment, female iNOS K.O. was bred with male WT C57BL6 on their matching ages. Their progeny (F1), iNOS heterozygote (iNOS Het), were bred (non brothers/sisters) and all males (20 iNOS K.O., 14 iNOS Het, and 15 WT littermates) were separated and genotyped for subsequent experiments. Mice were housed in groups of 4-5 per cage. Until the stress protocol started, all mice were kept in a quiet room with controlled temperature (24 ± 1°C), humidity (60 ± 5%), a dark-light cycle of 12h (lights on at 6:30 a.m.) with water and food *ad libitum*. To avoid acute stress due to the new environment, animals were allowed to acclimate for at least 1h to the experimental room throughout all the behavioral procedures.

### 2.2 Drugs

The following drugs were used: 5-Bromo-2’-deoxyuridine (BrdU; Sigma-Aldrich, MO, USA; 200 mg/Kg) (40) and escitalopram [Esc; Pratti-Donaduzzi, PR, Brazil; 10 mg/Kg - preprint study from our lab (41, 42)]. Ketamine and xylazine (both from Syntec, SP, Brazil; 150 mg/Kg and 8 mg/Kg, respectively) were used diluted in saline 0.9% as an anesthetic for tissue collection protocol.

### 2.3 Stress Protocol and Behavioral Tests

#### 2.3.1 Chronic Unpredictable Stress

WT and iNOS KO mice (outbred and inbred) were submitted to a randomized protocol of chronic unpredictable stress (CUS) for 21 consecutive days (43, 44). The CUS protocol consists of submitting the animals to one of the following daily stressors chosen randomly between the weeks to avoid habituation: forced swimming (15 minutes), restraint stress (2 hours), intermittent dark/light cycle (24h), inverted dark/light cycle (24h), wet bedding (24h), tilted cage (overnight) and food deprivation (24h) [adapted from (45)].

#### 2.3.2 Novelty-Suppressed Feeding Test

The animals were placed in a square acrylic box (50x50x40 cm) with the floor covered with sawdust. In the middle of the arena, a single pellet of food was placed on the top of a platform. Each animal was placed in one of the apparatus corners, and the latency to start feeding was measured with a cutoff period of 10 minutes. After the test, all animals returned to their home cages, and the amount of food consumed in a total period of 5 minutes was measured (43, 46).

#### 2.3.3 Tail Suspension Test

Animals were suspended by their tail at the height of 60cm and fixed by adhesive tape on a wooden surface. The experiment was recorded and the latency for the first immobile episode and time spent immobile during the 6 minutes was analyzed by an experimenter blinded to the animal experimental group. Animals that climbed or fell during the experimental session were excluded from the analysis (47).

#### 2.3.4 Open Field Test

The OFT was performed as a movement control to exclude any possible bias in NSFT and TST. The apparatus consisted of a circular arena with a diameter of 30cm. The test duration was 5 minutes and was performed in a sound-attenuated and temperature-controlled room. To analyze the total distance traveled, the ANYMAZE software (Stoelting Co, IL, USA) was used (18).

### 2.4 Behavioral Experiments Design

#### 2.4.1 Experiment 1: Effects Of Chronic Unpredictable Stress on iNOS KO Mice

**Figure 1:**
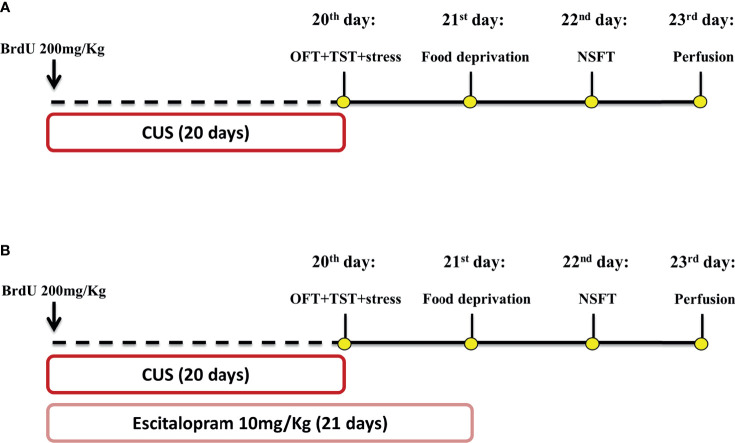
Schematic representation of treatment and stress protocol dynamic and behavioral assessment time course. Animals received a single dose of BrdU 200 mg/Kg 1h before the CUS protocol start. Independent experimental groups were treated with ESC 10 mg/Kg by 21 days **(B)** while the other remained without pharmacological manipulation **(A)**. CUS protocol lasted 20 days and was proceeded by OFT and TST tests on 20 day, food deprivation on the 21 day and NSFT on 22 day. All animals were submitted to the perfusion protocol and had their brain collected for posterior analysis **(A, B)**. This figure was created by one of the authors.

All animals received 1h before the first stressor 200 mg/Kg of BrdU, as illustrated in **Figure 1A**. Mice of all genotype groups were submitted to the CUS protocol or remained undisturbed on their home cage to serve as non-stressed controls. On the 20^th^ day of the experimental protocol, the animals were submitted to the OFT at 8 a.m., and subsequently to the TST at 1 p.m. On the 21^st^ day, all animals had their food removed, and after 24h were submitted to the NSFT. Twenty-four hours after the test, the animals were perfused and their brains removed for immunohistochemical assay (**Figure 1A**).

#### 2.4.2 Experiment 2: Effect of Escitalopram on iNOS KO Mice Submitted to CUS

As illustrated in **Figure 1B**, all animals received a single dose of BrdU (200 mg/kg), 1h before the first stressor. A randomized group of animals was submitted to the CUS protocol or remained undisturbed in their home cage. During CUS protocol, animals were treated with escitalopram (10mg/Kg) or vehicle from 4 p.m to 5 p.m. On the 20^th^ day of the experimental protocol, the animals were submitted to the OFT (experiments starting at 8 a.m) and the TST (experiments beginning at 1 p.m). On the 21^st^ day, all groups of animals had their food removed and after 20-24h were submitted to the NSFT. Twenty-four hours after the test, the animals were perfused and their brains removed for immunohistochemical assay (**Figure 1B**).

### 2.5 Immunoassays

#### 2.5.1 Tissue Preparation

The animals were deeply anesthetized with xylazine/ketamine perfused (through the introduction of a needle into the apex of the left ventricle) with 0.01M PBS followed by 4% PFA in 0.2 M PB for the immunoassays. The brains were removed and post-fixed for 24h in 4%PFA. The brains were transferred to a 30% sucrose solution for a week and then frozen on -30°C isopentane for cryoprotection. Eight to ten coronal slices with 30 µm thick were obtained from the prefrontal cortex (Bregma -2.1 to -1.5mm) and hippocampus (Bregma 1.46 to 3.08mm) using a cryostat at -20°C. The sections were kept in an anti-freezing solution (30% ethylene glycol/20% glycerol); until the assays were performed.

#### 2.5.2 Immunohistochemistry for Detection of DCX

Briefly, free-floating sections were washed three times with TBS (Tris Buffered saline, 50mM- 15 minutes each) followed by 30 min incubation with citrate buffer 10 mM (pH=6.0) at 70°C for antigen retrieval. After three washes with TBS (15 min/wash), the sections were incubated with a blocking solution (TBS + Triton X 0.025% + BSA 0.1%) for 4 hours followed by overnight incubation under agitation (18°C) with anti-DCX primary antibody (Santa Cruz Biotechnology, TX, USA; goat; 1:200). After three times washes with TBS, the sections were incubated for 1.5 h with a secondary antibody (Vectastain anti-goat biotinylated; 1:1000). Next, the complex A+B reaction was performed for 1h (1:500, ABC Elite-Vectastain kit, Vector Labs, Burlingame, CA, USA) followed by incubation with 3,3’-Diaminobenzidine (0.2mg/ml, 10 min, Sigma-Aldrich; MO, USA).

#### 2.5.3 Immunofluorescence for Detection of BrdU, DCX, and NeuN

After three washes with TBS, free-floating sections were incubated for 30 min with HCl 2N at 37°C, followed by two washes with boric acid (0.1M; pH=8.9; 10 min/wash) for antigen retrieval. The sections were incubated with the blocking solution (TBS + Triton X 0,025% + BSA 0.1%) for 4 hours, and then incubated with anti-BrdU (Abcam; Cambridge, England, UK; rabbit; 1:100), anti-DCX (Cell-Signaling; rat; 1:500) and anti-NeuN (Millipore; MA, USA; mouse; 1:500) primary antibodies overnight at 18°C under agitation. On the next day, the sections were washed three times with TBS-T (Tris-buffered saline with 10% Triton) and incubated for 1.5h with the secondary antibodies (anti-rabbit AlexaFluor 488, anti-rat Alexa-Fluor 594, and anti-mouse AlexaFluor 647; Invitrogen, Massachusetts, USA).

#### 2.5.4 Immunofluorescence for Detection of IBA-1, GFAP, SOX2, PV, and FOSB

As described in item 2.5.2, after the blocking section, slices were incubated with anti-IBA1 (Wako; VA; USA; rabbit; 1:1000) or with anti-GFAP (Cell-Signaling; MA; USA; mouse; 1:500) plus SOX2 (Millipore; MA, USA; rabbit; 1:500) or with anti-Parvalbumin (Sigma-Aldrich; MO, USA; mouse; 1:500) plus FOSB (Cell-Signaling; MA; USA; rabbit; 1:500). Then, sections were incubated for 1.5 h with secondary antibodies (anti-rabbit AlexaFluor 488, anti-mouse AlexaFluor 594; Invitrogen, Massachusetts, USA).

Hoechst staining (1:10,000 from 2µg/mL stock solution) was used to visualize and for the localization the layers and subregions of the prefrontal cortex and the hippocampal formation.

#### 2.5.6 Image Acquisition and Quantification

##### 2.5.6.1 Confocal Quantification of BrdU/DCX/NeuN, GFAP/SOX2, and PV/FosB

BrdU positive cells located at the granular layer of the dentate gyrus were visualized and manually quantified at 40X objective using Leica TSE-SPE microscope (Leica, Wetzlar, Germany). Confocal microscopy (Leica TSE-SPE) was performed for double and triple staining in the following sets: BrdU/DCX/NeuN, smart-gain:840V; GFAP/SOX2, smart-gain: 890V; PV/FOS, smart-gain: 950V. In all analyses, the smart offset was set as -100%, cells were tracked using z-stacks (6 steps) and visualized by 3D projections using the Leica X Software (Leica Microsystem, Wetzlar, Germany). For hippocampal staining, at least 6 slices containing both dentate gyri were analyzed. The absolute number of positive cells was calculated by the sum of the areas analyzed multiplied by the distance between them (43). For cortical staining, at least eight slices were analyzed, and the density of cells was calculated by dividing the number of positive cells and the area analyzed.

##### 2.5.6.2 IBA-1 Quantification and Sholl Analysis

IBA-1 positive cells were quantified on the granular layer of dentate gyrus using the epifluorescence microscope (Olympus, Shinjuku, Tokyo, Japan; 40X objective). The cell density was calculated by normalizing the number of cells counted by the area analyzed (measured using 10x objective). For Sholl analysis, IBA-1 positive cells located at the granular layer of the dentate gyrus were reconstructed using confocal microscopy (Leica TSE-SPE, objective 63X, smart-gain: 940V), and data were analyzed using Imaris Filament Tracer (v 9.3.1). Only cells presenting a whole labeling and visible process on 3D reconstruction were analyzed for their total process length and Sholl analysis parameter using concentric spheres of 5um radii. A total of 15 cells per animal were analyzed, totalizing 75 cells per group (32).

##### 2.5.6.3 Quantification of FOSB+ and PV+ Cells

For FOSB and PV cell quantification, images were acquired using the Leica TSE-SPE 20X objective. Positive cells were visualized and manually counted using Leica X Software (Leica Microsystem, Wetzlar, Germany). Values were normalized by the area analyzed using the same software.

### 2.6 Statistical Analysis

All data were analyzed for normal distribution using the Kolmogorov-Smirnov test and for homoscedasticity using Levene’s test before analysis. Data from inbred NSFT (**Supplement Figure 1D**) and SOX2/GFAP analysis (**Figure 5G**) did not follow these prerequisites. They were analyzed using the nonparametric Kruskal-Wallis and Mann-Whitney tests, with data represented as median and interquartile range. Otherwise, data collected from experiment 1 were analyzed using two-way ANOVA (factor 1: stress; factor 2: genotype), and data gathered from experiment 2 were analyzed using three-way ANOVA (factor 1: stress; factor 2: genotype; factor 3: treatment). When appropriated, Duncan’s *post hoc* test was performed. P values equal to or lower than 0.05 were considered significant. Data are represented as mean ± standard error of the mean (SEM).

## 3 Results

### 3.1 iNOS K.O. Mice Exhibit Decreased Survival Rates of Newborn Cells but No Changes in the Pattern of Microglial Activation in the DG

**Figure 2:**
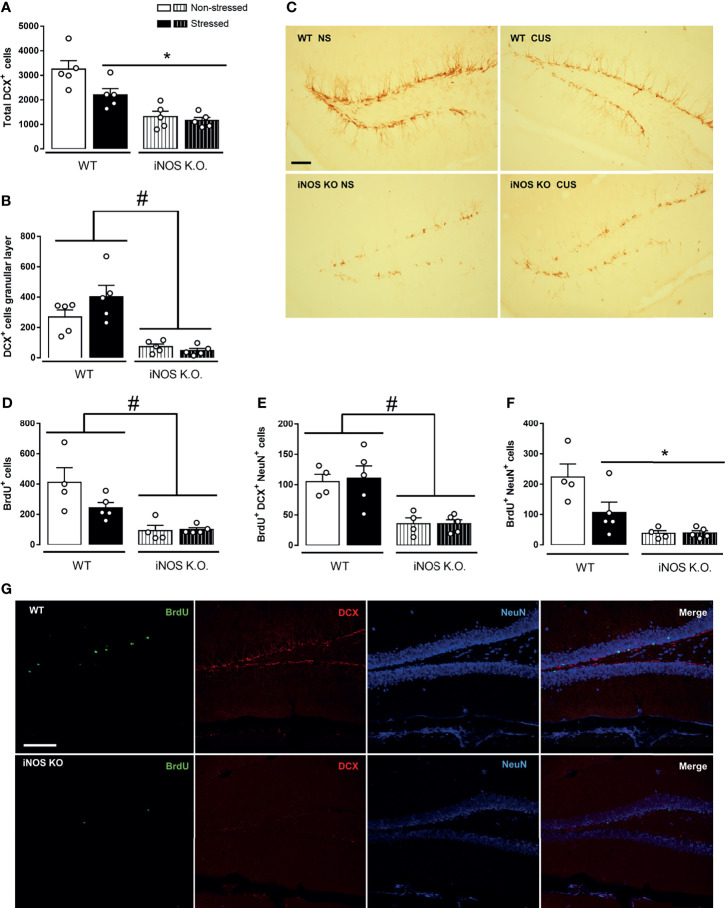
Adult-hippocampal neurogenesis is impaired on iNOS KO mice and does not exacerbate after CUS exposure. Quantification of neuroblast revealed a reduced number and migration towards the granular layer on iNOS KO mice that did not alter after CUS [**(A, B)**, respectively]. Representative image of neuroblast (DCX+ cells) on the dentate gyrus of all groups analyzed (n=5/group, scale bar = 100 µm) **(C)**. BrdU incorporation analysis revealed a reduced survival of adult-born new neurons on iNOS KO mice that was not affected by CUS [**(D–F)**; n = 4,5,4 and 5 respectively]. Representative images of immunofluorescence technique showing BrdU, DCX NeuN and Merge of markers on WT non-stress and iNOS KO non-stressed group (scale bar = 100 µm) **(G)**. Data present as mean ± SEM * indicates p < 0.05 compared to WT NS mice. # indicates p < 0.05 on genotype factor.

Initially, we investigated neurogenic parameters in iNOS KO under control or submitted to CUS. iNOS K.O. mice present a lower number of neuroblasts expressing doublecortin (DCX+ cells - classified as Type IIb and III). Different from WT animals, CUS did not affect the number of DCX+ cells (2 way-ANOVA df=1,19; genotype: F=35.94, p<0.05; stress: F=5.87, p<0.05; interaction: F=3.20; **Figure 2A**). As these cells migrate towards the granular layer to maturate and integrate the regional circuitry, we assessed the number of DCX+ present on the granular layer. Corroborating our previous observation, iNOS K.O. mice presented a reduced number of DCX+ on the granular layer which was not affected by CUS exposure (Two way-ANOVA df=1,19; genotype: F=36.84, p<0.05; stress: F=1.36; interaction: F=3.06; **Figures 2B, C**).

Next, we accessed the survival rate of adult newborn neurons in WT and iNOS K.O mice by evaluating the total number of BrdU+ cells 23 days after BrdU administration. Our results showed that iNOS K.O. mice exhibit a reduced number of BrdU+ cells on the dentate gyrus (not further diminished by CUS) (Two way-ANOVA df=1,17; genotype: F=21.97, p<0.05; stress: F=2.61; interaction: F=3.15; **Figure 2D**). We also analyzed the phenotype of BrdU+ cells by performing a triple-staining analysis (BrdU+DCX+NeuN). Cells classified as type IIb/III (BrdU+DCX+NeuN+) were reduced in iNOS K.O. mice (Two way-ANOVA df=1,17; genotype: F=21.99, p<0.05; stress: F=2.65; interaction: F=3.16; **Figure 2E**). In the same direction, we observed that mature neurons (BrdU+NeuN+) were reduced on iNOS K.O. mice and unaffected by CUS. Interestingly, CUS reduced the survival of adult newborn neurons on WT mice (Two way-ANOVA df=1,17; genotype: F=20.87, p<0.05; stress: F=4.40, p=0.054; interaction: F=4.63, p<0.05; **Figure 2F**). Representative images of each marker are found in **Figure 2G**.

**Figure 3:**
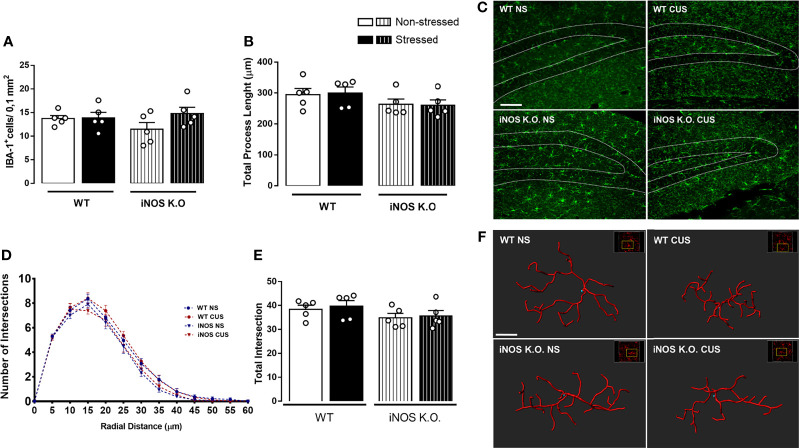
Chronic unpredictable stress does not alter microglia density **(A)** or complexity **(C–E)** on the granular zone of the dentate gyrus neither of WT or iNOS KO mice **(B)**. Representative photomicrography of dentate gyrus of all groups analyzed (scale bar = 99,98 µm) and filament 3D reconstruction on IMARIS (scale bar = 7 µm) **(F)**. Data presented as mean ± SEM (n=5/group).

As microglia cells are resident cells of the hippocampal neurogenic niche (25, 48). Thus, we investigated if their density and morphology in the granular layer of the DG would be affected in WT or iNOS K.O mice submitted to CUS (32, 49). CUS did not affect the microglia density in the dentate gyrus (Two way-ANOVA df=1,19; genotype: F=0.32; stress: F=2.16; interaction: F=1.80; **Figure 3A**). Regarding their morphological features (**Figure 3C**), none of our manipulations modified their process length (Two way-ANOVA df=1,19; genotype: F=3.66; stress: F=0.03; interaction: F=0.47; **Figure 3B**). Sholl’s analysis revealed that there were no differences regarding their interaction with 5µm radii spheres (**Figure 3D**) and total intersections (Two way-ANOVA df=1,19; genotype: F=3.45; stress: F=0.25; interaction: F=0.01; **Figure 3E**).

### 3.2 CUS Does Not Modify the Behavioral Phenotype of iNOS K.O. Observed in Control/Basal Conditions

**Figure 4:**
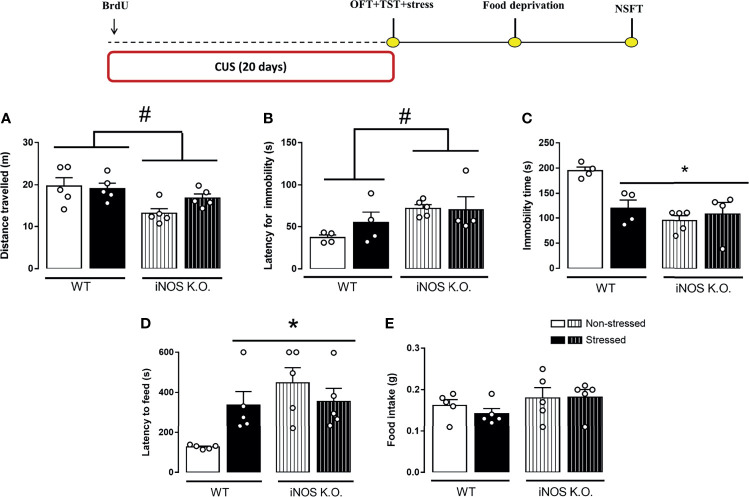
Distinct behavioral response to CUS of outbred WT and iNOS K.O. mice. **(A)** total distance traveled on the open field test (n=5/group); latency for the first immobility episode **(B)** and total immobility time on the tail suspension test (n=4,4,5 and 4, respectively) **(C)**; **(D)** latency to feed in a novel environment **(E)**: total feeding consumption during five minutes in home-cage (n=5/group). Data presented as mean ± S.E.M. *p < 0,05 from WT non-stressed; # indicates p < 0.05 on genotype factor.

Using different breeding strategies, we observed that outbred iNOS K.O. mice had an increased latency for the first immobility episode that was unaffected by CUS (Two way-ANOVA df=1,16; genotype: F=6.52, p<0.05; stress: F=0.64; interaction: F=0.95; **Figure 4B**). Also, outbred iNOS K.O. mice presented an active coping response in the TST that is different from WT mice, was not affected by CUS (Two way-ANOVA df=1,16; genotype: F=14.07, p<0.05; stress: F=4.27; interaction: F=8.58, p<0.05; **Figure 4C**). Regarding anxiety-related behaviors, iNOS K.O. mice presented an anxiogenic-like phenotype which, different from the WT animals, was not affected by CUS exposure (Two way-ANOVA df=1,19; genotype: F=7.81, p<0.05; stress: F=1.45; interaction: F=4.52, p<0.05; **Figure 4D**). None of the interventions changed the food intake in the home cage (Two way-ANOVA df=1,19; genotype: F=2.57; stress: F=0.38; interaction: F=0.23; **Figure 4E**). To access their locomotor activity, which may affect the TST response, we performed the OFT test. iNOS K.O. mice presented an increased locomotor activity compared to WT mice (Two way-ANOVA df=1,19; genotype: F=9.71, p<0.05; stress: F=0.49; interaction: F=1.29; **Figure 4A**).

### 3.3 Escitalopram Did Not Rescue AHN Parameters in iNOS K.O. Mice

**Figure 5:**
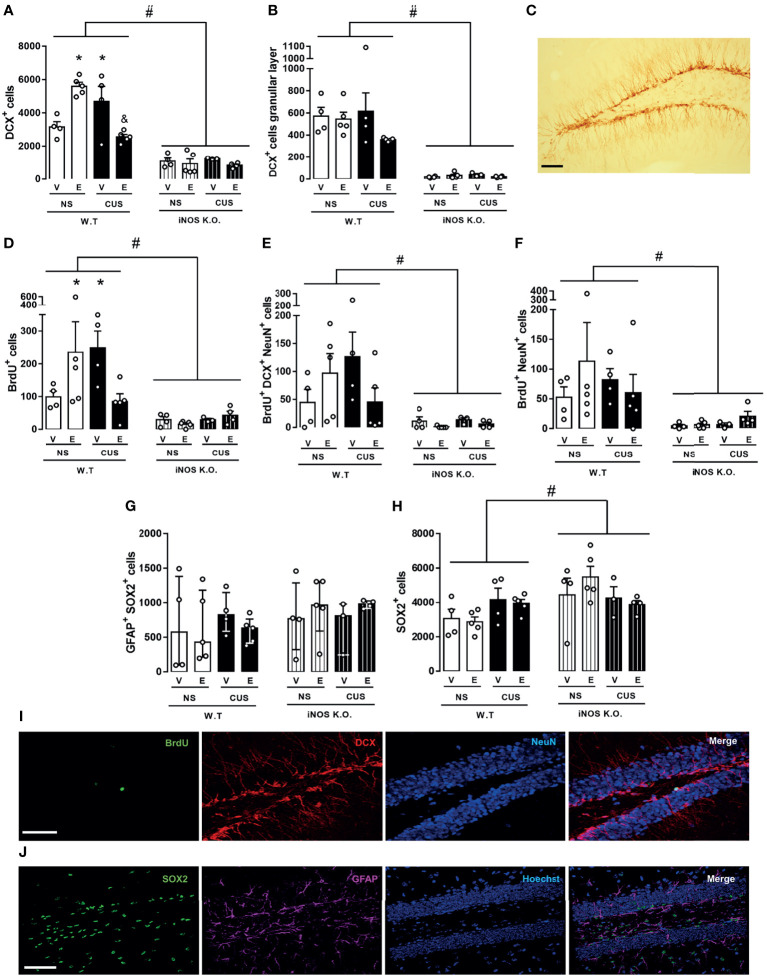
Escitalopram does not modify the AHN impairment observed on iNOS K.O. mice. Quantification of neuroblast total number and migration towards the granular layer [**(A, B)**, respectively]. BrdU incorporation analysis revealed a reduced survival of adult-born new neurons on iNOS KO mice that was not affected by escitalopram or CUS interventions [**(D–F)**; n=4,5,4,5,4,5,3 and 4, respectively]. Type I cells analysis showed that these cells are not altered on iNOS KO mice, nor was affected by any manipulations employed **(G)**. Quantification of SOX2 positive cells [**(H)**; n=4,5,4,5,4,5,3 and 4, respectively]. Representative image showing DCX, BrdU/DCX/NeuN and GFAP/SOX2 positive cells in the dentate gyrus of a WT non-stressed animal [**(C, I, J)**, respectively; scale bar = 50 µm]. Data present as mean ± SEM **(A, B, D–F, H)** or median and interquartile range **(G)** * indicates p<0.05 compared to WT NS mice. # indicates p < 0.05 on genotype factor, & p < 0.05 compared with WT CUS vehicle mice.

Next, we investigated whether chronic escitalopram treatment, a pharmacological pro-neurogenic stimulus, would rescue the reduced neurogenic profile of iNOS K.O. mice. Escitalopram did not facilitate AHN in iNOS K.O. mice. In the WT animals, escitalopram and, surprisingly, CUS augmented the number of DCX+ cells in the dentate gyrus. This effect was not observed when these two conditions were combined (Three way-ANOVA df=1,33; genotype: F=128.56, p<0.05; stress: F=1.98; treatment: F=0.05; genotype x stress: F= 2.18; genotype x treatment: F= 0.70; stress x treatment: F= 20.93, p<0,05; stress x treatment x genotype: F= 16.87, p<0,05 **Figure 5A**). Also, iNOS K.O. mice presented a lower number of migrating cells manipulations compared with WT mice, as independent effect of stress or drug treatment (Three way-ANOVA df=1,33; genotype: F=108.78, p<0.05; stress: F=0.45; treatment: F=2.34; genotype x stress: F= 0.68; genotype x treatment: F= 2.15; stress x treatment: F= 1.89; stress x treatment x genotype: F= 1.05, **Figure 5B**).

We also addressed the question whether the BrdU+ incorporation and retention in adult newborn neurons in iNOS k.O mice would be rescued by escitalopram (**Figure 5I**). Similar to our DCX observations, none of our interventions induced any significant change in iNOS K.O. mice. In WT animals, escitalopram and CUS increased the survival of cells that incorporated BrdU in the dentate gyrus. The effect was independent of stress and drug treatment (Three way-ANOVA df=1,33; genotype: F=18.15, p<0.05; stress: F=0.40; treatment: F=0.14; genotype x stress: F= 0.48; genotype x treatment: F= 0.02; stress x treatment: F= 5.17, p<0.05; stress x treatment x genotype: F= 5.58, p<0.05 **Figure 5D**). To characterize the fate of BrdU cells, we investigated the number of type IIb/III cells colocalized with BrdU. None of our interventions promoted any significant changes in either iNOS K.O. or WT mice (Three way-ANOVA df=1,33; genotype: F=15.85, p<0.05; stress: F=0.27; treatment: F=0.44; genotype x stress: F= 0.12; genotype x treatment: F= 0.01; stress x treatment: F= 3.41; stress x treatment x genotype: F= 3.58 **Figure 5E**). In the same direction, none of our interventions changed the number of mature adult-born neurons in WT or iNOS K.O. mice (Three way-ANOVA df=1,33; genotype: F=8.64, p<0.05; stress: F=0.01; treatment: F=0.35; genotype x stress: F= 0.22; genotype x treatment: F= 0.06; stress x treatment: F= 0.55; stress x treatment x genotype: F= 1.11, **Figure 5F**).

Lastly, we questioned whether our results would reflect an impaired neurogenic niche capacity, reflected by reduced numbers of neural stem cells or type I cells (GFAP+SOX2+ cells) located at the subgranular zone. To answer this question, we measured the number of GFAP+SOX2+ cells in all our interventions (**Figure 5J**). Surprisingly, none of our interventions modified the number of type I cells in the dentate gyrus of our animals (χ2 = 4,59). The Type IIa cells (SOX2+), also denominated intermediate progenitor cells or transient amplifying progenitors cells, possess a high mitotic activity (2). Their number increased in iNOS K.O. mice. None of our interventions modified this number (Three way-ANOVA df=1,33; genotype: F=6.44, p<0.05; stress: F=0.52; treatment: F=0.02; genotype x stress: F= 0.62; genotype x treatment: F= 0.46; stress x treatment: F= 0.83; stress x treatment x genotype: F= 0.75, **Figure 5H**).

### 3.4 The Chronic Treatment With Escitalopram Failed to Rescue the Antidepressant And Anxiogenic Phenotype of iNOS K.O. Mice

**Figure 6:**
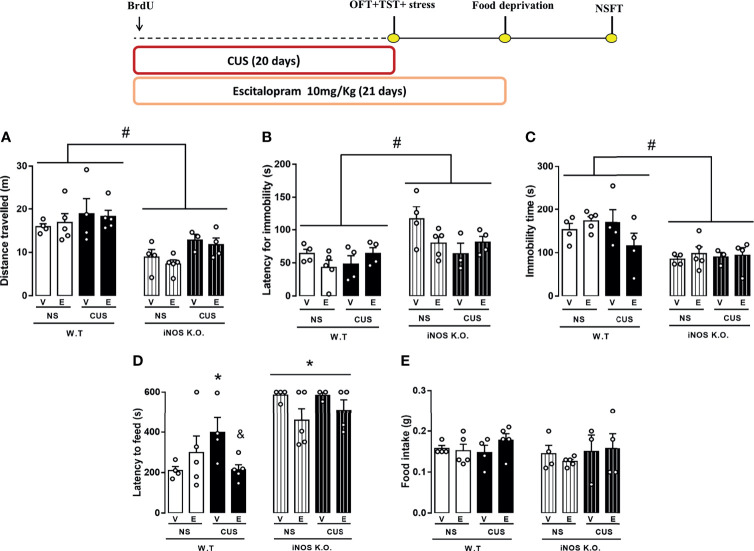
Escitalopram is devoid of behavioral effects on iNOS KO mice. **(A)** total distance travelled on the open field test (n=4,5,4,5,4,5,3 and 4 respectively); **(B)** first immobility episode and **(C)** total immobility time on the tail suspension test (n=4,5,4,4,4,4,3 and 4 respectively); **(D)** latency to feed in a novel environment **(E)** total feeding consumption during five minutes in home-cage (n=4,5,4,5,4,5,3 and 4 respectively). Data presented as mean ± S.E.M. *p < 0,05 from WT non-stressed vehicle; # indicates p < 0.05 on genotype factor; & p < 0.05 compared with WT CUS vehicle mice.

Our observations suggested that in the TST,the latency to the first immobility was not changed compared to their respective controls (3 way-ANOVA df=1,32; genotype: F=13.01, p<0.05; stress: F=1.87; treatment: F=0.54; genotype x stress: F= 2.87; genotype x treatment: F= 0.18; stress x treatment: F= 0.30; stress x treatment x genotype: F= 0.25.11, **Figure 6B**). On the same direction, none of our interventions modified the total immobility time in the TST (Three way-ANOVA df=1,32; genotype: F=20.83, p<0.05; stress: F=0.54; treatment: F=0.08; genotype x stress: F= 0.62; genotype x treatment: F= 0.88; stress x treatment: F= 2.46; stress x treatment x genotype: F= 1.48, **Figure 6C**). In the NSFT, CUS augmented whereas Escitalopram treatment decreased the latency to feed on the novel environment in the WT mice (Three way-ANOVA df=1,33; genotype: F=42.97, p<0.05; stress: F=0.99; treatment: F=3.68; genotype x stress: F= 0.12; genotype X treatment: F= 0.47; stress X treatment: F= 2.08; stress x treatment x genotype: F= 4.40, p<0.05, **Figure 6D**). No changes in the behavioral profile of iNOS mice was found in the NSFT. There was no food intake difference in the home-cage consumption test (Three way-ANOVA df=1,33; genotype: F=1.08; stress: F=0.94; treatment: F=0.71; genotype x stress: F= 0.17; genotype x treatment: F= 0.35; stress X treatment: F= 1.08; stress X treatment X genotype: F= 0.03, **Figure 6E**). The OFT showed that iNOS K.O. mice keep presenting an hypolocomotion compared to WT mice and neither CUS protocol nor ESC treatment could change this behavior (Three way-ANOVA df=1,33; genotype: F=42.97, p<0.05; stress: F=0.99; treatment: F=3.61; genotype x stress: F= 0.12; genotype X treatment: F= 0.47; stress x treatment: F= 2.08; stress x treatment x genotype: F= 3.48, **Figure 6A**).

### 3.5 The CUS Decreased, But ESC Restored, the Chronic Activation of Prelimbic Portions of the vmPFC Observed in iNOS K.O. Mice

**Figure 7:**
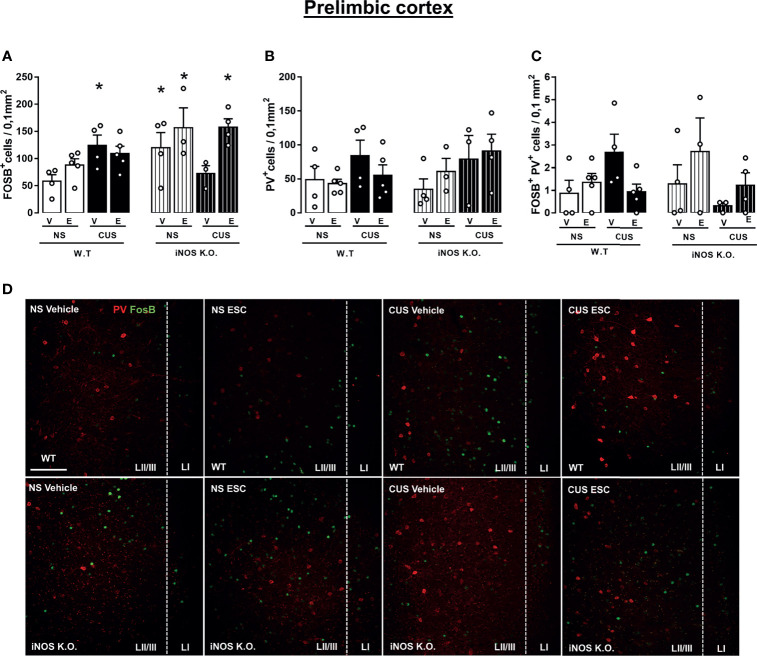
Escitalopram does not modify the parvalbumin-positive cells recruitment on prelimbic cortex (PrL) of iNOS KO mice. FOSB^+^ cells quantification on PrL **(A)**, PV+ cells density on PrL **(B)** and FOSB^+^PV^+^ colocalization density on PrL and IL **(C)**. Representative images showing FOSB+ and PV+ cells of all experimental groups analyzed (**D**; scale bar = 50 µm). N=4,5,4,5,4,3,3,4, respectively. *p < 0,05 from WT non-stressed vehicle.

Lastly, we investigated if the cellular activation and the number of parvalbumin (PV+) interneurons in the subregions of the ventromedial prefrontal cortex (vmPFC). CUS increased cellular activation in the prelimbic (PrL) area in WT animals that was not modified by escitalopram. iNOS KO mice also presented and increased number of FOSB+ cells in the prelimbic (PrL). In this case, this difference was attenuated by CUS exposure. In these animals (iNOS K.O. exposed to CUS), escitalopram increased the number of FOSB+ cells. (Three way-ANOVA df=1,31; genotype: F=5.55, p<0,05; stress: F=0.57; treatment: F=6.43, p<0.05; genotype x stress: F= 6.07, p<0.05; genotype x treatment: F= 3.95; stress x treatment: F= 0.01; stress x treatment x genotype: F= 2.99, **Figure 7A**). The density of PV+ cells was not different among the groups (Three way-ANOVA df=1,31; genotype: F=0.38; stress: F=3.81; treatment: F=0.01; genotype x stress: F= 0,23; genotype x treatment: F= 1,71; stress x treatment: F= 0.45; stress x treatment x genotype: F= 0.02, **Figure 7B**).We also did not observed any increase of the activation of PV+ cells by co-labeling with FOSB in the PrL (Three way-ANOVA df=1,31; genotype: F=0.02; stress: F=0.32; treatment: F=0.31; genotype x stress: F= 4.03; genotype x treatment: F= 3.53; stress x treatment: F= 2.06; stress x treatment x genotype: F= 0.79, **Figures 7C, D**).

## 4 Discussion

In the present study, we demonstrated that that iNOS K.O. mice exhibit impaired survival of newly generated neurons in the adult DG (**Figure 2**). Moreover, manipulation to either reduce (CUS) or augment AHN (chronic escitalopram treatment) failed to produce their classical behavioral effects in mice (7,47 - **Figures 2** and **4**). The behavioral phenotypes of K.O. mice are also not modified by these manipulations (stress or antidepressant treatment **Figures 3** and **5**). Together, these findings suggest that iNOS may be an essential factor for the survival of adult newborn neurons and behavioral adaptability. The behavioral changes observed in homozygosis is also present in heterozygous iNOS+/- mice (**Supplement Figure 1**). Even though homozygosis related to a genetic absence of iNOS has been described in humans, this condition seems to be fatal when individuals are exposed to pathogens. This observation indicates that our results could be relevant in the context of human health since heterozygosis of INOS gene may exist among the human population (50), especially regarding the behavioral adaptability and its correlation with the incidence of mental disorders.

The involvement of nitric oxide synthase enzymes in the process of AHN was also described by Carreira and colleagues (2015). Using a different experimental design, the authors showed that administration of kainic acid, a drug able to induce seizures and artificially induced neural precursor cell proliferation in mice, lacks the latter property in animals lacking the iNOS (51, 52). In the same direction, genetic ablation of iNOS also prevents ischemia-induced neurogenesis in rodents (53). Besides, it has been shown that neonatal iNOS KO mice presented the same retention of BrdU on the SGZ as WT mice, highlighting that the former observations, as well as ours, may not be associated with an impaired hippocampal development (22).

Several lines of evidence support the hypothesis that the microglia cell phenotype differently modulates the AHN (28). For instance, systemic administration of lipopolysaccharide induces recruitment and activation towards an inflammatory phenotype of microglial cells, leading to reduced survival of adult newborn-neuron (27). On the other hand, Ziv and colleagues (2006) showed that the pro neurogenic effects induced by environment enrichment depend on T-cells and, ultimately, the recruitment of microglia present in the neurogenic niche (54). Mechanistically, it has been proposed that NO and reactive oxidative species (ROS) produced by the microglia cells are the mediators of their detrimental effects on AHN neurogenesis (55, 56). Supporting this assumption, Carreira and colleagues (2014) showed that inflammatory stimulus reduced the proliferation of neural stem cells derived from the subventricular zone only when these cells were cocultured with microglia that expressed iNOS (23). Also, Tang and colleagues (2018) showed that chronic social defeat stress (SDS), a well-known model that diminishes AHN (57), induces the microglia cells to express iNOS in the dentate gyrus. In this study, however, the authors did not explore whether this effect is related to the SDS behavioral outcome (34).

Despite the evidence mentioned above, we observed that iNOS K.O. mice had an impaired AHN (**Figure 2**). Moreover, no changes in microglial cells were observed in the dentate gyrus (**Figure 3**). This latter result contrasts with several reports showing that chronic stress, either homotypic or heterotypic, affect microglia cells in limbic structures resulting in changes in their density, morphology, and priming status (30–33, 58–60). One factor that could help to explain this discrepancy is that some studies did not restrict their observation to one subregion of the dentate gyrus. For instance, Kreisel and colleagues (2014) analyzed the entire dentate gyrus (30). On the other hand, Hellwig and colleagues (2016) restricted their morphological analysis to the inferior molecular layer of the dentate gyrus (32). Even if our results suggest that our CUS protocol did not modify microglial cells in the dentate gyrus, they need to be confirmed by other molecular markers such as the expression of MHC-II, CD-68 (associated with pro-inflammatory microglia response), and the presence of phagocytic calyxes (morphological feature related to phagocytosis of apoptotic new-born cells) (25, 61).

In parallel to our AHN and microglial observations, we also report that iNOS K.O. mice present an antidepressant- and anxiogenic-like phenotype not altered by CUS exposure or chronic escitalopram treatment. Previous studies from the literature had already shown that pharmacological or genetic iNOS modulation facilitates an active coping strategy on the forced swim test (18). Also, the administration of a selective iNOS inhibitor, 1400W, prevented anhedonic-like behavior induced by CUS (19). Concerning anxiety-like behaviors, iNOS K.O. mice presented an anxious-like phenotype and enhanced freezing in the fear conditioning paradigm (62, 63).

The present data also suggests a possible mechanist explanation for this behavioral profile. Several studies indicate that AHN work as fine-tune mechanism for the hippocampal function, especially those related to spatial navigation and stress adaptability [for a recent review, see Surget and Belzung (11)]. Corroborating this proposal, impaired AHN in our study was associated with an anxiogenic response in the NSFT (**Figure 3**) and a lack of response after chronic treatment with escitalopram (**Figure 5**). These observations are in line with those made by Santarelli and co-workers (2003) where they suggest that the behavioral effects of antidepressants depend on intact AHN (44). Genetic manipulations aiming at facilitating the AHN process is associated with antidepressant- and anxiolytic-like effect on animals chronically exposed to corticosterone (64). However, other studies using a similar genetic approach to enhance neurogenesis failed to reproduce the previous results on a CUS paradigm, suggesting that the behavioral properties of AHN may be related to the challenge and protocol of stress used (6, 65). Our results also determine that the survival of adult newborn neurons observed in iNOS K.O. mice was not related to an impaired neurogenic niche in our study. Corroborating these results, Arnhold and colleagues (2002) showed that pharmacological inhibition of iNOS impairs the proper neuronal development and differentiation of embryonic neural stem cells (66).

Our results also suggested a dichotomy between the behavioral effects of escitalopram and possible facilitation on adult hippocampal neurogenesis (by means of DCX+ cells). Our hypothesis is that this apparently contradictory results might relies on the interpretation of DCX+ cells as measures of newborn neurons survival and migration (67). In fact, Plümpe and colleagues (2006) showed that the expression of DCX in the dentate gyrus is independent of the regulation of precursor cell proliferation (68). Together these finds might indicate that in our conditions escitalopram could facilitate AHN *via* other cell markers. In addition, study conducted in postmorten brain samples of suicide patients, an increased expression of DCX and higher volume of their process in the subventricular zone/olfactory bulb pathways (69). Although reported in a different neurogenic niche, their results indicate that the link between adult neurogenesis, DCX and stress-related disorders is far from clear and deserve further investigation. Of course, we cannot rule out the participation of other mechanisms, such as dendritic spine remodeling on limbic structures (70, 71) and perineuronal-net assembly and maintenance (72, 73) could also be involved in the behavioral effects of escitalopram repeated treatment.

Because our results regarding iNOS and AHN only partially explained the behavioral alterations found in iNOS K.O mice we decide to investigate the involvement of the vmPFC. Changes in vmPFC activity have been demonstrated in preclinical models and in patients diagnosed with major depressive and anxiety disorders (74). Moreover, high-frequency stimulation of this region is capable of promoting structural changes on the hippocampus, including enhanced AHN (75–78).

It was recently proposed that fast act-antidepressants could act by interfering with a distinct population of interneurons present in the vmPFC, including PV+ cells (37, 79–81). Specific manipulations of PV interneurons in the vmPFC recapitulate the behavioral response observed after a single dose of ketamine, a fast-acting antidepressant/anxiolytic drug (37). Corroborating these observations, Page and colleagues (2019) showed that after 4 weeks of CUS exposure, stressed-mice presented augmented cFos reactivity (a marker of neuronal activation) in PV+ cells in the vmPFC (35).

FOSB expression has been associated with cellular activation after repeated stimuli (38). In agreement with our results, different chronic stress protocols increase the expression and accumulation of FOSB in the vmPFC (82, 83). Even if our results indicate that this effect does not depend on a specific increase in PV+ cells, additional studies are needed to investigate: i) whether the augmentation reported here occurs on GAD67+ cells or excitatory neurons; and ii) whether other interneurons populations, such as neurons expressing somatostatin (37), are recruited on iNOS K.O. mice.

Also, it is needed to take into consideration that we did not evaluate the central levels of inflammatory cytokines, which have been implicated with psychiatric disorders (84). In the same vein, there are pieces of evidence that suggest that iNOS K.O. mice express a basal cytokine profile that resembles those observed in WT animals (85, 86). Specifically, Cummings and Tarleton showed that unstimulated iNOS K.O. mice splenocyte presented similar secretion levels of interferon-γ, tumor necrosis factor-α, interleukin 1 (IL-1), Il-6, granulocyte-macrophage colony-stimulating factor, and CCL-3 (previously known as macrophage inflammatory protein-α). Moreover, it has been shown that iNOS K.O. mice present similar plasmatic levels of IL-1β to the wild-type mice, and iNOS KO mice-derived bone-marrow macrophages present similar nuclear protein levels of NF-kB and IkKα to the WT (87). As NF-kB is a critical player on inflammatory processes, regulates expression of multiple cytokines and on astrocytes is an important regulatory gene that mediates secretion of BDNF and NGF (88, 89), our results seem to reflect that iNOS rather than other inflammatory mediators is the critical component of our observations. Also, our genetic manipulation promoted the total absence of iNOS making it difficult to unearth which cell may be the most influential on the phenotype observed. In this sense, further studies aiming to selectively ablated iNOS on specific glial populations, such as microglia and astrocytes, are still needed.

Taken together, our observations suggest that in iNOS K.O. mice that AHN modulates anxiety-related behaviors after stress, while the PrL cortex could be involved in depressive-like behaviors (74, 90).

## 5 Conclusion

iNOS seems to play an essential role in the survival of newborn cells and immature neurons in the hippocampus. In addition, the lack of this enzyme appears to result in increased activity of the PrL prefrontal cortex. These two parallel neurobiological mechanisms could help to explain the antidepressant and anxiogenic behavioral profile observed in the iNOS KO mice. Whereas impaired AHN would increase anxiety, a higher PrL activity would be involved with positive copying behavior strategies. Further studies with site-directed conditional knockouts and direct pharmacological interventions are needed to address these possibilities.

## Data Availability Statement

The original contributions presented in the study are included in the article/**Supplementary Material**. Further inquiries can be directed to the corresponding author.

## Ethics Statement

The animal study was reviewed and approved by the Experimental Committee of the University of São Paulo.

## Authors Contributions

GF conceptualized the project, performed the behavioral and immunohistochemical procedures, analyzed the data, and wrote the draft of the manuscript. KC performed behavioral and immunohistochemical procedures, reviewed the figures, and helped to write the first draft of the manuscript. DS performed genotype experiments, performed behavioral procedures, and reviewed the first draft of the manuscript. JF participated in the immunohistochemical experiments and their analysis. FG revised the manuscript. AC was the project principal investigator, conceptualized the project, provided the resources, reviewed, and edited the draft versions of the manuscript. All authors approved the final version of the manuscript before its initial submission.

## Funding

This study was supported by the FAPESP Young research grant (2015/05551-0), the thematic grant (2017/24304-0), the L’Oreal-Unesco-Brazilian Academy of Science for Women in Science Fellowship, and CNPq Universal Grant line A (400033/2016-0). The funder was not involved in the study design, collection, analysis, interpretation of data, the writing of this article or the decision to submit it for publication. AC and FG are recipients of CNPq research fellowships. GF and JF received scholarships from CNPq. DS and KC are CAPES fellowships.

## Conflict of Interest

The authors declare that the research was conducted in the absence of any commercial or financial relationships that could be construed as a potential conflict of interest.

## Publisher’s Note

All claims expressed in this article are solely those of the authors and do not necessarily represent those of their affiliated organizations, or those of the publisher, the editors and the reviewers. Any product that may be evaluated in this article, or claim that may be made by its manufacturer, is not guaranteed or endorsed by the publisher.
