# Supplementary material for: Genetic Ablation of the Inducible Form of Nitric Oxide in Male Mice Disrupts Immature Neuron Survival in the Adult Dentate Gyrus
Source: Front Immunol. 2021 Dec 1;12:782831. doi: 10.3389/fimmu.2021.782831 (PMC8673740; doi:10.3389/fimmu.2021.782831)
Supplement: Supplementary file 1 [file DataSheet_1.docx]

**Supplement: Behavioral effects of CUS on inbred iNOS K.O. mice.**

Regarding the inbred mice, none of our manipulations altered the first immobility episode (Two way-ANOVA df=1,46; genotype: F=0.70; stress: F=0.01; interaction: F=1.32; Supplementary Fig1B). We observed an impact of stress in WT and iNOS K.O. mice (Two way-ANOVA df=1,46; genotype: F=0.29; stress: F=7.16; interaction: F=0.32; Supplementary Fig1C). In the NSFT, the Kruskal-Wallis test revealed a difference among the groups (χ^2^= 11.63, p<0.05). iNOS K.O. mice tended to present an anxiogenic-like phenotype not altered by CUS (Mann-Whitney WT NS x iNOS NS= 20, p=0.07; WT NS x iNOS CUS= 10, p<0.05; iNOS NS x iNOS CUS= 38). WT mice tended to increase their latency to feed in the novel environment (Mann-Whitney WT NS x WT CUS= 11.5, p=0.051; Supplementary Fig1D). None of the interventions changed food intake in home cage (Two way-ANOVA df=1.48; genotype: F=2.22; stress: F=1.14; interaction: F=0.64; Supplementary Fig 1E). The OFT revealed that none of our interventions modified the locomotor activity of the animals (Two way-ANOVA df=1,48; genotype: F=0.14; stress: F=2.66; interaction: F=1.89; Supplementary Fig 1A).


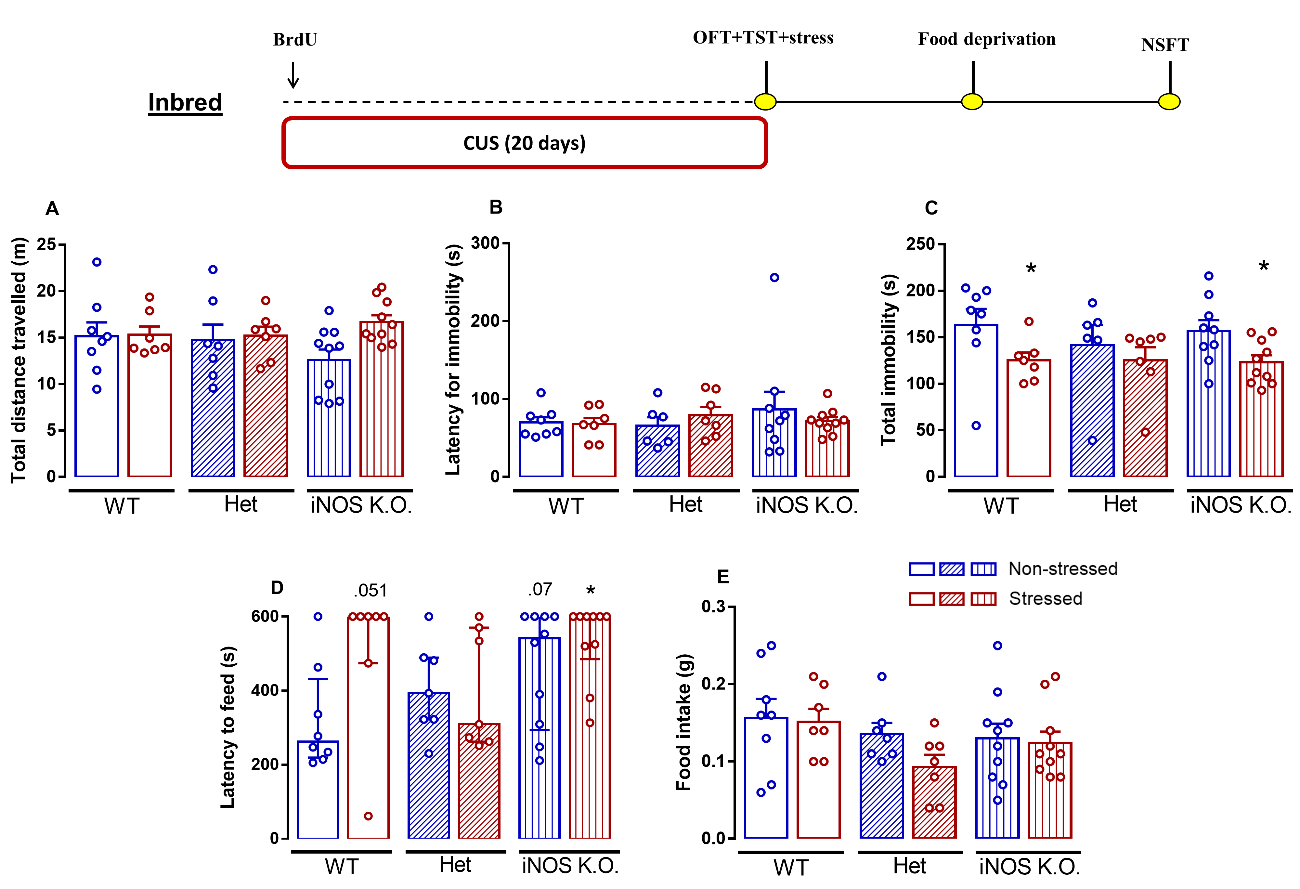


Supplementary 1: Distinct behavioral response to CUS of inbred WT and iNOS K.O. mice. (A): total distance traveled on the open field test (n=8, 7, 7, 7, 10, and 10, respectively); latency for the first immobility episode (B) and total immobility time on the tail suspension test (n= 8, 7, 6, 7, 9, and 10, respectively) (C); (D): latency to feed in a novel environment (E): total feeding consumption during five minutes in home-cage (n=8, 7, 7, 7, 10, and 10, respectively). Data presented as mean ± SEM (A, B, C, E) or median and interquartile range (D). *p<0,05 from WT non-stressed.

**Supplement: CUS and escitalopram does not modified activation of infralimbic portion of the vmPFC.**

CUS and escitalopram did not promote a significant change in the density of FOSB+ cells in the infralimbic (IL) cortex (Three way-ANOVA df=1,31; genotype: F=0.26; stress: F=0.07; treatment: F=2.71; genotype x stress: F= 1,65; genotype x treatment: F= 1.31; stress x treatment: F= 2.87; stress x treatment x genotype: F= 0.15, Supp. Fig 2A), nor on PV+ cells density (Three way-ANOVA df=1,31; genotype: F=0.14; stress: F=2,90; treatment: F=0.35; genotype x stress: F= 0.10; genotype x treatment: F= 3.29; stress x treatment: F= 0.37; stress x treatment x genotype: F= 0.06, Supp. Fig 2B). Also, none of the manipulations induced significant changes on FOSB+PV+ cells on IL cortex (Three way-ANOVA df=1,31; genotype: F=1.55; stress: F=1.16; treatment: F=0.14; genotype x stress: F= 0.04; genotype x treatment: F= 1.11; stress x treatment: F= 0.11; stress x treatment x genotype: F= 0.01, Supp. Fig 2C).


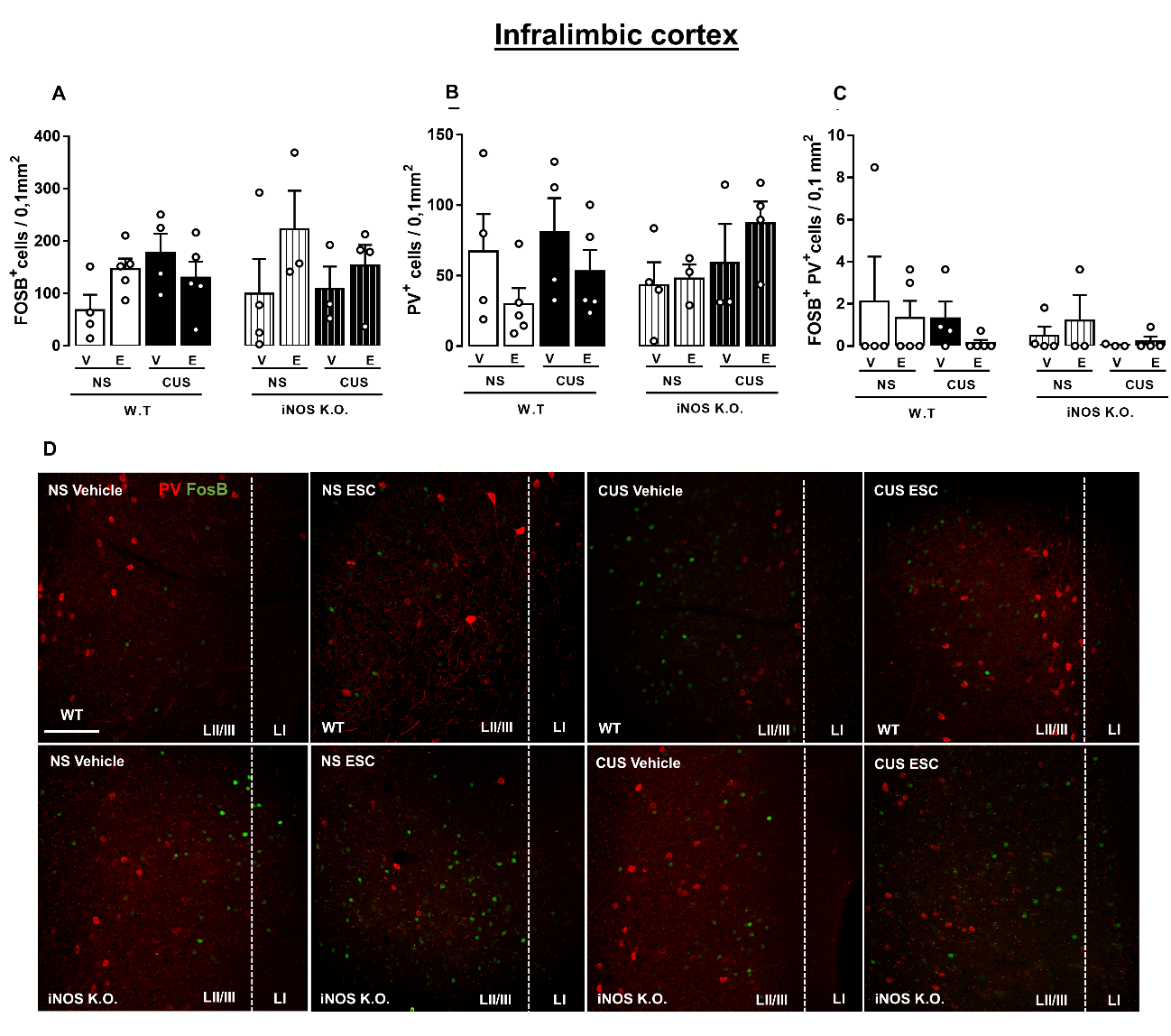


Supplementary 2: Escitalopram does not modify the parvalbumin-positive cells recruitment on infralimbic cortex of iNOS KO mice. FOSB^+^ cells quantification on IL (A), PV+ cells density on IL (B) and FOSB^+^PV^+^ colocalization density on IL (C). N=4,5,4,5,4,3,3,4, respectively.
