# Supplementary figures and images for: Genetic Ablation of the Inducible Form of Nitric Oxide in Male Mice Disrupts Immature Neuron Survival in the Adult Dentate Gyrus
Source: Front Immunol. 2021 Dec 1;12:782831. doi: 10.3389/fimmu.2021.782831 (PMC8673740; doi:10.3389/fimmu.2021.782831)

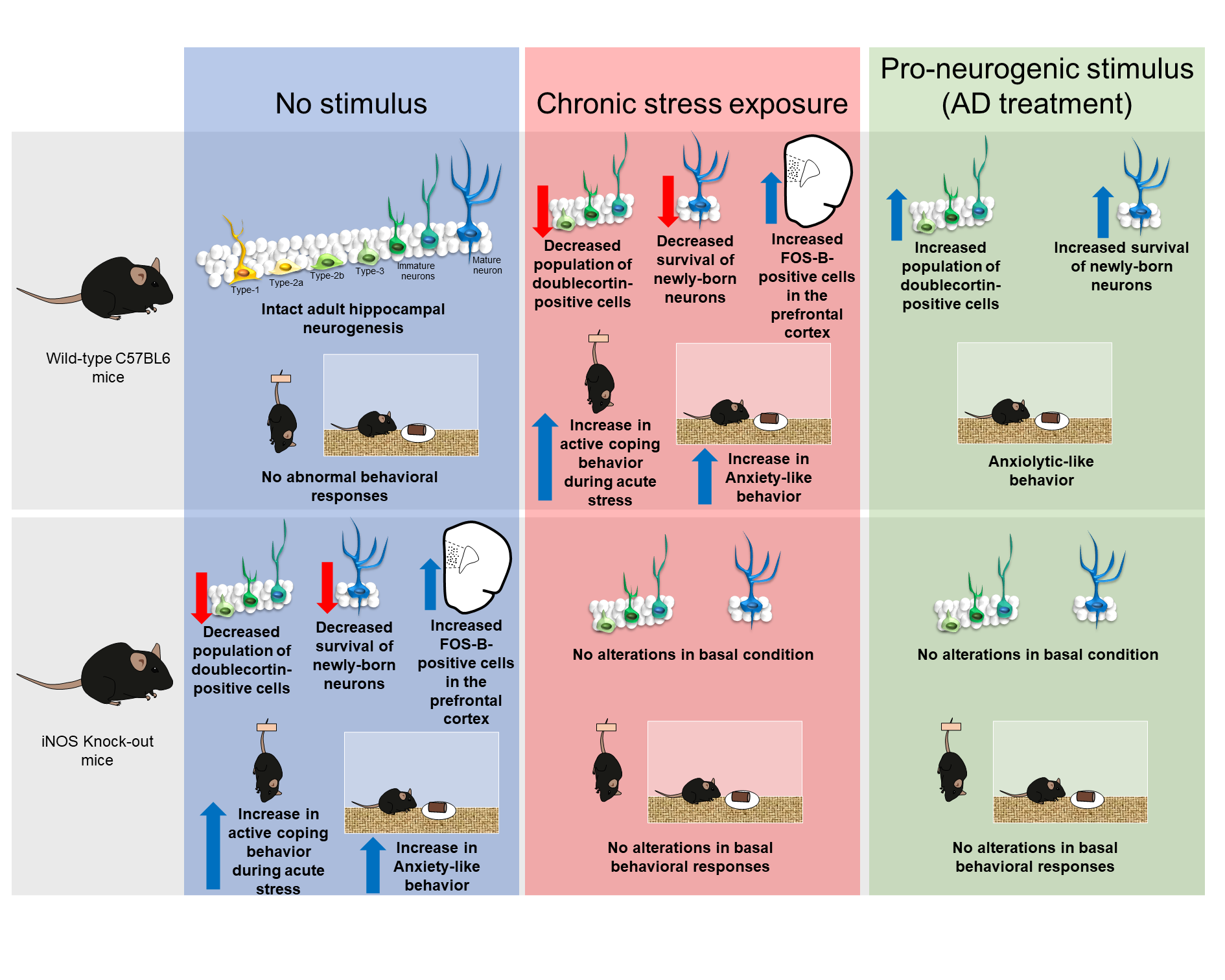

Supplement: Supplementary file 2 [file Image_1.tif]
